# Supplementary material for: Nutrition Meets Social Marketing: Targeting Health Promotion Campaigns to Young Adults Using the Living and Eating for Health Segments
Source: Nutrients. 2021 Sep 10;13(9):3151. doi: 10.3390/nu13093151 (PMC8470224; doi:10.3390/nu13093151)
Supplement: Supplementary file 1 [file nutrients-13-03151-s001.zip › nutrients-1364178-supplementary.pdf]

Supplementary Table S1: Charactristics of measures as reported in Table 2, 3, and 4. (https://doi.org/10.26180/5dba10f4ec6e5)

**Characteristic**	**Question**	**Scoring Criteria**	**Maximum Score**	**Items**
Cause of obesity – Energy balance	How much do you agree with the following statements?	5-point Likert scale<br>1 – Strongly disagree<br>5 – Strongly agree	10	(1) Obesity is usually caused by overeating<br>(2) Obesity is usually caused by lack of exercise
Cause of obesity – Medical	How much do you agree with the following statements?	5-point Likert scale<br>1 – Strongly disagree<br>5 – Strongly agree	10	(1) Obesity can be caused by a biological disorder<br>(2) Food can be like an addiction for some people (e.g. sugar), just like drugs
Cause of obesity – Willpower	How much do you agree with the following statements?	5-point Likert scale<br>1 – Strongly disagree<br>5 – Strongly agree	5	(1) Obesity is rarely caused by a lack of willpower
Self-perception of cooking	How much do you agree/disagree with the following statements?	5-point Likert scale<br>1 – Strongly disagree<br>5 – Strongly agree	35	(1) I am a good cook<br>(2) Others view me as a good cook<br>(3) I am a relatively better cook than my family/friends<br>(4) I don't consider myself to be a good cook (Reverse)<br>(5) I get a sense of satisfaction from preparing and cooking meals<br>(6) I am confident that whatever I cook will turn out well<br>(7) I can time different elements of a dish to come together on time
Choosing healthy foods	How much do you agree/disagree with the following statements?	5-point Likert scale<br>1 – Strongly disagree<br>5 – Strongly agree	5	(1) I am very particular about the healthiness of the food I eat
Meal planning & prepping food	How good would you say you are at the following tasks:	5-point Likert scale<br>1 – Very Poor<br>5 – Very Good	15	(1) Planning meals ahead (e.g. for the day/week ahead)<br>(2) Following recipes when cooking<br>(3) Preparing meals in advance e.g. packed lunch, partly preparing a meal in advance
Shopping	How good would you say you are at the following tasks:	5-point Likert scale<br>1 – Very Poor<br>5 – Very Good	15	(1) Planning how much food to buy<br>(2) Shopping with a grocery list<br>(3) Shopping for specific meals
Budgeting	How good would you say you are at the following tasks:	5-point Likert scale<br>1 – Very Poor<br>5 – Very Good	20	(1) Knowing what budget you have to spend on food<br>(2) Comparing prices before you buy food<br>(3) Buying food in season to save money<br>(4) Buying cheaper cuts of meat or fish to save money

Characteristic	Question	Scoring Criteria	Maximum Score	Items
Label comprehension and use	How good would you say you are at the following tasks:	5-point Likert scale<br>1 – Very Poor<br>5 – Very Good	20	(1) Reading the storage and use by information on food packets<br>(2) Reading nutrition information on food labels<br>(3) Balancing meals based on nutrition advice of what is healthy<br>(4) Reading the best-before date on food
Resourcefulness	How good would you say you are at the following tasks:	5-point Likert scale<br>1 – Very Poor<br>5 – Very Good	25	(1) Keeping basic ingredient items in your cupboard for putting meals together e.g. herbs/spices, dried/tinned goods<br>(2) Preparing or cooking a healthy meal with only few ingredients on hand<br>(3) Preparing or cooking a meal with limited time<br>(4) Using leftovers to create another meal<br>(5) Cooking more or double recipes which can be used for another meal
Subjective norms to healthy eating	How much do you agree/disagree with the following statements?	5-point Likert scale<br>1 – Strongly disagree<br>5 – Strongly agree	15	(1) My family think I should eat healthier<br>(2) My close friends think I should eat healthier<br>(3) My health practitioner(s) think I should eat healthier
Motivation to comply	How much do you agree/disagree with the following statements?	5-point Likert scale<br>1 – Strongly disagree<br>5 – Strongly agree	15	(1) Generally speaking, I do what my family expects of me<br>(2) Generally speaking, I do what my close friends expect of me<br>(3) Generally speaking, I do what my health practitioner(s) expects of me
Current satisfaction with healthy eating	How much do you agree/disagree with the following statements?	5-point Likert scale<br>1 – Strongly disagree<br>5 – Strongly agree	15	(1) I currently eat healthily<br>(2) I am satisfied with the healthiness of my diet<br>(3) I don't need to improve the healthiness of my diet
Positive attitudes towards healthy eating	How much do you agree/disagree with the following statements?	5-point Likert scale<br>1 – Strongly disagree<br>5 – Strongly agree	10	(1) It is important that the food I eat keeps me healthy<br>(2) Following a healthy diet is a good thing to do
Heathy eating is difficult	How much do you agree/disagree with the following statements?	5-point Likert scale<br>1 – Strongly disagree<br>5 – Strongly agree	5	(3) It is too difficult to follow a healthy diet
Self-efficacy	How much do you agree/disagree with the following statements?	5-point Likert scale<br>1 – Strongly disagree<br>5 – Strongly agree	15	(1) I can solve most problems if I invest the necessary effort<br>(2) If I am in trouble, I can usually think of a solution<br>(3) I can usually handle whatever comes my way

Characteristic	Question	Scoring Criteria	Maximum Score	Items
Perceived nutrition knowledge/expertise	How much do you agree/disagree with the following statements?	5-point Likert scale<br>1 – Strongly disagree<br>5 – Strongly agree	20	(1) I know quite a bit about healthy eating<br>(2) I do not feel very knowledgeable about healthy eating<br>(3) When it comes to healthy eating, I really don't know a lot<br>(4) Compared to most people, I know less about healthy eating
Nutrition knowledge Mavenism	How much do you agree/disagree with the following statements?	5-point Likert scale<br>1 – Strongly disagree<br>5 – Strongly agree	10	(1) In my circle of friends, I am one of the "experts" on healthy eating<br>(2) People seek me out for information on healthy eating
Intention towards healthy eating	How much do you agree/disagree with the following statements?	5-point Likert scale<br>1 – Strongly disagree<br>5 – Strongly agree	15	(1) I intend to improve to healthiness of my diet over the next month<br>(2) I plan to eat a healthier diet over the next month<br>(3) I want to eat a healthier diet over the next month
Unhealthy eating habits (skipping breakfast during week)	On average in the past month on weekdays (Mondays to Fridays only), how often do you do the following?	5-point Likert scale<br>1 – Almost never<br>5 – Almost everyday	5	(1) Skip breakfast (e.g. don't eat anything within 2 hours of waking up)
Unhealthy eating habits (dinner before bed during week)	On average in the past month on weekdays (Mondays to Fridays only), how often do you do the following?	5-point Likert scale<br>1 – Almost never<br>5 – Almost everyday	5	(1) Eat dinner within two hours before bedtime
Unhealthy eating habits (skipping breakfast during weekend)	On average in the past month on weekends (Saturdays and Sundays only), how often do you do the following?	5-point Likert scale<br>1 – Almost never<br>5 – Almost everyday	5	(1) Skip breakfast (e.g. don't eat anything within 2 hours of waking up)
Unhealthy eating habits (dinner before bed during weekend)	On average in the past month on weekends (Saturdays and Sundays only), how often do you do the following?	5-point Likert scale<br>1 – Almost never<br>5 – Almost everyday		(1) Eat dinner within two hours before bedtime

Characteristic	Question	Scoring Criteria	Maximum Score	Items
Quality of Life EUROHIS	Please rate the following questions based on your satisfaction levels.	5-point Likert scale<br>1 – Very poor/dissatisfied<br>2 – Very good/satisfied	40	(1) How would you rate your quality of life?<br>(2) How satisfied are you with your health?<br>(3) How would you rate your energy for everyday life?<br>(4) How satisfied are you with yourself?<br>(5) How satisfied are you with your living conditions?<br>(6) How satisfied are you that you have enough money to meet your needs?<br>(7) How satisfied are you with your ability to perform your daily living activities?<br>(8) How satisfied are you with your personal relationships?
Quality of Life Meiselman	Please rate the following questions based on your satisfaction levels.	5-point Likert scale<br>1 – Very poor/dissatisfied<br>2 – Very good/satisfied	20	(1) How satisfied are you with what you are achieving in life?<br>(2) How satisfied are you with feeling part of your community?<br>(3) How satisfied are you with your future security?<br>(4) How satisfied are you with your spirituality or religion?
